# Lung carcinoma with adrenal metastasis and inferior vena cava thrombosis in an elderly patient with decompensated chronic liver disease: a case report

**DOI:** 10.1097/MS9.0000000000002459

**Published:** 2024-08-14

**Authors:** Pramodman Singh Yadav, Abinash Dev, Leeza Shah, Ashish Khadka, Pratik Adhikari, Arash Pyakurel

**Affiliations:** a Department of Internal Medicine, B.P. Koirala Institute of Health Sciences, Dharan; b Department of Internal Medicine, Chitwan Medical College, Bharatpur; c Department of Radiodiagnosis and Imaging, National Academy of Medical Sciences, Kathmandu; d Department of Internal Medicine, Birat Medical College and Teaching Hospital, Tankisinuwari, Nepal

**Keywords:** anticoagulation, case report, malignancy, metastasis, palliative, thrombus

## Abstract

**Introduction:**

Managing patients with complex comorbidities poses significant diagnostic and therapeutic challenges. This case report details a 65-year-old male with a history of decompensated chronic liver disease (CLD) and portal hypertension, who presented with symptoms suggestive of liver disease exacerbation. He was later diagnosed with primary lung malignancy and extensive thrombosis, including the inferior vena cava (IVC) and heart chambers, a rare finding.

**Case presentation:**

A 65-year-old man with a history of smoking, alcohol consumption, and chronic liver disease presented with severe pain in the upper right quadrant, dyspnea, weakness, loss of appetite, and unintentional weight loss. Medical assessments revealed decompensated CLD with elevated bilirubin levels, low albumin, and an elevated INR. Imaging showed lung cancer with metastasis to the adrenal gland and a large IVC thrombus extending to the heart chambers. The patient decided to pursue palliative care.

**Discussion:**

When dealing with primary lung cancer and adrenal metastasis, it’s important to thoroughly assess atypical presentations for IVC thrombus. Even with advances in imaging and treatments, managing IVC thrombus related to cancer is still difficult and requires a team approach. This case highlights underdiagnosis in areas with limited resources, emphasizing the need for timely advanced diagnostics such as CT and MR imaging.

**Conclusion:**

This case highlights the complexities of diagnosing and managing patients with multiple conditions. It emphasizes the need for patient-centered care and the importance of ongoing research to develop effective diagnostic and treatment strategies for conditions like IVC thrombus in the context of malignancy.

## Introduction

Highlights

- IVC thrombus is rare in Nepal due to healthcare limits and lower VTE risk in Asians.
- Sixty-five-year-old with CLD, lung cancer, adrenal metastasis, and extensive IVC thrombosis.
- CT and MR imaging are vital for identifying thrombus and malignancies in atypical cases.
- Treatment involves heparin bridging, long-term warfarin; advanced cases may need surgery.
- Patient’s choice for palliative care highlights the importance of autonomy in treatment.

In Asian countries like Nepal, the incidence of inferior vena cava (IVC) thrombus is generally low, potentially as a result of resource constraints in healthcare settings, and a relatively lower risk of venous thromboembolism (VTE) in ethnic Asian populations^1^. Tumor thrombus is an uncommon complication of solid tumors and is typically observed as either a metastatic tumor deposit or as a tumor extension into a vein^2^. Furthermore, IVC thrombus secondary to primary lung carcinoma is a rare finding, and when present may also involve the pulmonary vein, left atrium, and heart chambers^3,4^. This case report details a 65-year-old male with underlying risk factors for chronic liver disease (CLD) who initially presented with manifestations suggestive of decompensated CLD. However, further investigation unveiled a more complex pathology: primary lung malignancy with metastasis to the adrenal gland, complicated by extensive thrombosis involving the IVC, hepatic veins, and extension into the right atrium and ventricle (RA/RV). This rare finding is associated with IVC thrombus, hence the report’s importance. This report has been reported in line with the CARE criteria^5^. IJS Patient Consent Form has been used to obtain consent from the patient and is available to the journal upon request.

Virchow’s triad—comprising stasis of blood flow, endothelial injury, and hypercoagulability—forms the cornerstone of venous thromboembolism pathophysiology, including IVC thrombosis^6^. Among the plethora of etiologies that can lead to VTE, malignancy contributes to ~17% of cases, followed by IVC anomalies, and venous compression^1^. Malignancy significantly increases the risk of VTE, with cancer patients having a seven-fold higher risk compared to non-cancer populations^7^. Cancer accounts for 37.5% of IVC thrombus cases, demonstrating a strong relationship between malignancy and IVC thrombus, and the most common cancer associated is renal cell carcinoma (38%), followed by other genitourinary cancers (25%)^8,9^.

The symptoms of IVC thrombosis can vary and may include leg heaviness, pain, swelling, and cramping, as well as nonspecific symptoms like abdominal, flank, or back pain. Men may experience scrotal swelling. Diagnosis can be delayed until clot migration to the lungs and renal veins occurs, resulting in shortness of breath and oliguria. Additionally, bilateral lower extremity edema and dilated superficial abdominal veins may suggest the possibility of an IVC thrombus^10^. Overall, there are no specific history and physical examination findings to consistently diagnose this condition across all patients due to its ambiguous presentation^11^. The association of IVC thrombus with secondary lower limb deep vein thrombosis (DVT), pulmonary embolism (PE), and a heightened risk of thromboembolic events, particularly in predisposing conditions such as CLD and previous malignancies can be another important clue to its suspicion^12^.

When IVC thrombosis is suspected, carefully reviewing the lower extremity duplex ultrasound is crucial. This test can provide vital information about potential blockages higher up the vein, even if there’s no evidence of DVT in the lower leg. However, it’s important to consider that ultrasound results can vary based on the operator’s skill, and visualizing the IVC can be challenging due to factors like bowel gas or obesity. Therefore, timely computed tomography (CT) and magnetic resonance (MR) imaging are also essential for an accurate diagnosis^13,14^. Treatment typically involves anticoagulation therapy, commonly initiated with heparin bridging followed by long-term warfarin administration^15^.

## Case presentation

A 65-year male, active smoker (20 pack-year), past alcohol consumer, known case of Decompensated CLD and portal hypertension, hypothyroidism with a history of recurrent upper gastric ulcer bleed presented to the emergency department with a two-day history of severe dull aching right upper quadrant (RUQ) pain that had progressed over 6 months. He also reported progressive shortness of breath of unspecified duration on exertion relieved by sitting, generalized weakness, loss of appetite, and unintentional weight loss. The patient had a significant history of alcohol abuse (locally brewed beverage, 500 ml/day for 30 years) and tobacco chewing for 40 years. He did not have a history of any surgical procedure. On examination, the patient displayed pallor, bilateral lower limb edema, icterus of unspecified severity, and lymphadenopathy in the cervical and axillary regions, along with increased BMI. His blood pressure was recorded as 140/90 mm Hg upon presentation. Cardiovascular examination revealed jugular venous distention (JVD) measuring 8 cm H2O, and normal heart sounds (S1 and S2) were detected without murmurs. The respiratory assessment showed tachypnea at 22 breaths per minute and bibasilar crackles. Abdominal examination revealed tenderness in the right upper quadrant (RUQ) and a palpable liver ~6 cm below the right costal margin, with dullness on percussion. The neurological examination was also grossly intact, but a flapping tremor was present in both hands. There was no history of unilateral calf pain, redness, or swelling of the limb. There was no history of suggestive of any abdominal/lung mass. Upon presentation to the emergency department, relevant investigations were promptly initiated (Table 1), and the patient was managed conservatively with the following treatments: intravenous pantoprazole at 40 mg once daily, intravenous ceftriaxone at 1 g twice daily, and intravenous Buscopan at 20 mg as needed for pain. Additionally, intravenous fluids were administered to maintain hydration and support hemodynamic stability.

**Table 1:** Laboratory findings

Parameter	Result	Reference range
Prothrombin time (PT)	13 sec	9.5–13.8 sec
INR	1.97	0.8–1.2
Total bilirubin	3.1 mg/dl	0.1–1.2 mg/dl
Direct bilirubin	2.0 mg/dl	0–0.3 mg/dl
Albumin	2.6 g/dl	3.5–5.0 g/dl
Renal function tests	Normal	Normal

INR, international normalized ratio.

**Figure 1:**
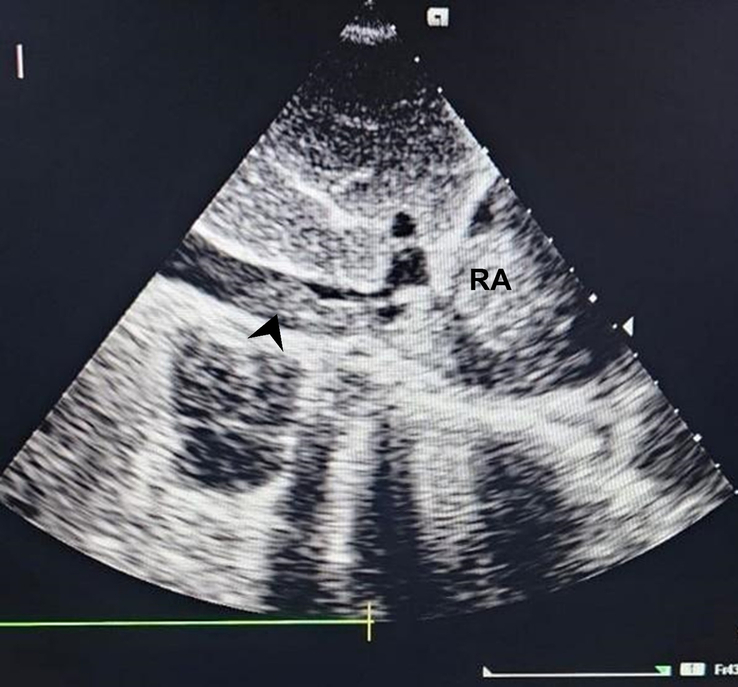
Transthoracic echocardiography—an echogenic mass protruding from the inferior vena cava (black arrowhead in the figure) into the right atrium (RA).

**Figure 2:**
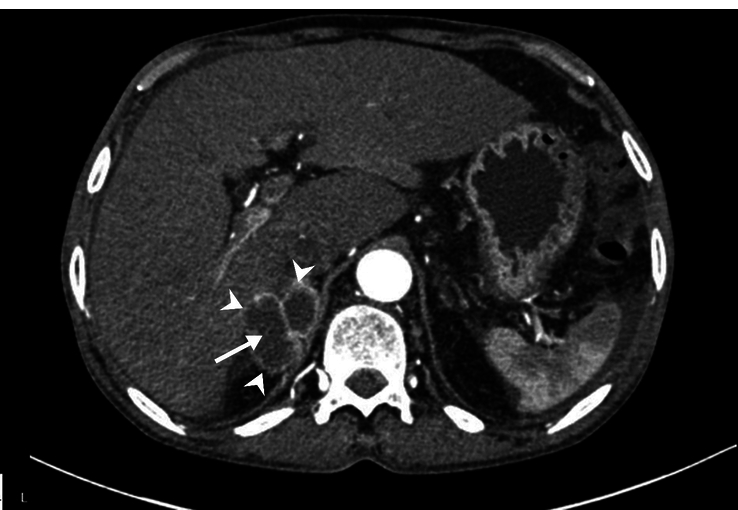
Contrast-enhanced computed tomography of the abdomen and pelvis demonstrates a large, heterogeneous mass in the right adrenal gland with areas of necrosis (white arrow) and peripheral enhancement (white arrowhead), suggestive of an adrenal tumor.

**Figure 3:**
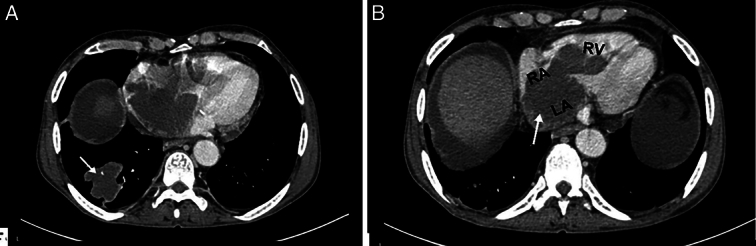
Contrast-enhanced computed tomography of the chest demonstrates right primary lung malignancy [white arrow in (A)], and IVC thrombus extending into the left atrium (LA), right atrium (RA), and right ventricle (RV) [white arrow in (B)].

The patient was then admitted to the gastroenterology ward for further evaluation and management. Given his significant weight loss, palpable lymph nodes, and transthoracic echocardiographic findings (an echogenic mass protruding from the IVC into the RA and RV (Fig. 1), a comprehensive workup including a contrast-enhanced computed tomography (CECT) scan was performed. A CECT of the abdomen and pelvis identified an adrenal tumor, presenting as a large, heterogeneous mass in the right adrenal gland with areas of necrosis and peripheral enhancement, suggestive of an adrenal tumor (Fig. 2). Additionally, CECT of the chest demonstrated a primary lung malignancy (Fig. 3A). Furthermore, it identified an IVC thrombus, presenting as an extensive thrombus extending into the left atrium, right atrium, and right ventricle (Fig. 3B). Table 2 summarizes the radiological findings of the case.

**Table 2:** Radiological findings

Imaging modality	Findings
Transthoracic echocardiography	Figure 1: Transthoracic echocardiography—an echogenic mass protruding from the inferior vena cava (black arrowhead in the figure) into the right atrium (RA)
CECT of abdomen and pelvis	Figure 2: CECT of the abdomen and pelvis demonstrates a large, heterogeneous mass in the right adrenal gland with areas of necrosis (white arrow) and peripheral enhancement (white arrowhead), suggestive of an adrenal tumor
CECT of chest	Figure 3: CECT of the chest demonstrates right primary lung malignancy [white arrow in (A)], and IVC thrombus extending into the left atrium (LA), right atrium (RA), and right ventricle (RV) [white arrow in (B)]

CECT, contrast-enhanced computed tomography; IVC, inferior vena cava.

A multidisciplinary discussion involving gastroenterologists, oncologists, and radiologists was held. A provisional diagnosis of lung malignancy metastatic to the right adrenal gland with decompensated CLD and extensive thrombosis involving the IVC, hepatic veins, and RA/RV was established. The patient was counseled regarding further investigations and treatment options, including potential aggressive therapies. However, the patient declined further aggressive therapy and opted for palliative care.

## Discussion

This case report highlights a rare and complex case. This report’s comprehensive diagnostic approach and multidisciplinary management enhance its credibility. It emphasizes the importance of individual quality-of-life considerations in clinical decision-making. However, as a single case report, the findings cannot be generalized, and the lack of long-term follow-up and genetic or molecular analysis limits its insights. The report also notes potential underdiagnosis in resource-constrained settings and highlights the influence of reporting bias on the objectivity of the findings.

In some cases, the presence of an extensive tumor thrombus secondary to primary lung carcinoma is a rare but critical finding, often detected using F-18 FDG PET-CT imaging modalities^16^. This case reveals the atypical presentation of moderately differentiated adenocarcinoma with concurrent hepatic and adrenal metastases from the primary lung tumor. Metastasis to the adrenal glands, particularly from lung primaries, underscores the aggressive nature of certain malignancies. Adrenocortical carcinoma with extension into the IVC via the adrenal vein is exceptionally rare and typically associated with a poor prognosis^17^.

The rarity of adrenal gland involvement in metastatic disease highlights its significance as a less common yet impactful site of spread. Hematogenous dissemination through the adrenal veins, leading to IVC involvement, remains a dominant pathway, although direct extension from large tumors or lymphatic spread can also contribute^18^. The anatomical features of adrenal veins, including potential discrepancies in size between the right adrenal vein (RAV) and left adrenal vein (LAV), may influence the propensity for IVC metastasis^18^. Adrenocortical carcinoma (ACC) exhibiting non-functioning characteristics, as observed in this case, is often clinically silent hormonally, manifesting predominantly through local mass effects such as abdominal discomfort, nausea, and vomiting^19^.

Management strategies for advanced non-small cell lung cancer (NSCLC) with adrenal and IVC involvement typically involve a multimodal approach to optimize outcomes. In our case, proposed treatment strategies included neoadjuvant immunotherapy with pembrolizumab and carboplatin to reduce tumor burden and enhance resectability^20^. Surgical resection aimed at complete removal of the right adrenal gland and subsequent adjuvant radiation therapy were considered to mitigate the risk of recurrence^21^. However, the patient’s decision for palliative care reflects the importance of aligning treatment choices with individual preferences and quality-of-life considerations^22^. Yet, it is important to keep in mind that the identification of an IVC tumor thrombus poses significant challenges in management, often necessitating specialized surgical intervention or anticoagulant therapy such as rivaroxaban to mitigate thromboembolic risks^23^.

## Conclusion

Managing a case with primary lung cancer, extensive metastasis, and thrombosis is challenging. Identifying an IVC thrombus extending into the right atrium and ventricle, compounded by adrenal metastasis, underlines the need for comprehensive diagnostic evaluations. Treating IVC thrombus secondary to cancer necessitates a multidisciplinary approach. The patient’s preference for palliative care over aggressive treatment emphasizes patient-centered care. This case highlights the potential for underdiagnosis of conditions in resource-constrained settings and emphasizes the need for advanced diagnostic tools such as CT and MR imaging.

## Ethical approval

Not applicable.

## Consent

Informed consent was taken from the patient to publish this case report.

## Source of funding

Not applicable.

## Author contribution

A.D.: writing of original draft of the manuscript, resources, review and editing, conceptualization, editing, data curation, visualization, investigator. P.M.S.Y.: resources, validation, project administration, review and editing, supervision. L.S.: resources, data curation, investigator, project administration, review and editing, supervision. A.K.: validation, images, supervision. P.A.: review and editing, supervision. A.P.: review and editing, supervision.

## Conflicts of interest disclosure

The authors declare no conflict of interest.

## Research registration unique identifying number (UIN)

This is a case report, so registration was not required.

## Guarantor

Abinash Dev is the guarantor of the study.

## Data availability statement

The datasets supporting the conclusions of this article are included within the article.

## Provenance and peer review

Not commissioned or externally peer-reviewed.
